# Supplementary material for: Impact of the Regularization Parameter in the Mean Free Path Reconstruction Method: Nanoscale Heat Transport and Beyond
Source: Nanomaterials (Basel). 2019 Mar 11;9(3):414. doi: 10.3390/nano9030414 (PMC6473983; doi:10.3390/nano9030414)
Supplement: Supplementary file 1 [file nanomaterials-09-00414-s001.pdf]

Impact of the regularization parameter in the Mean Free Path reconstruction method: Nanoscale heat transport and beyond

M.A. Sánchez-Martínez,[1] F. Alzina, [1] J. Oyarzo, [2] C.M. Sotomayor-Torres,[1, 3] and E. Chavez-Angel[*1]

[1)] Catalan Institute of Nanoscience and Nanotechnology (ICN2), CSIC and The Barcelona Institute of Science and Technology (BIST), Campus UAB, Bellaterra, 08193 Barcelona, Spain.

[2)] Instituto de Química, Pontificia Universidad Católica de Valparaíso, Casilla 4059, Valparaíso, Chile.

[3)] ICREA- Institució Catalana de Recerca i Estudis Avançats, 08010 Barcelona, Spain.

*Corresponding author: emigdio.chavez@icn2.cat

Magnon-mediated longitudinal spin-Seebeck effect in YIG films

This data was obtained by Guo *et al* measuring the thickness dependence of the longitudinal-Spin Seebeck effect (LSSE) in YIG films.[1] The suppression function used in the reconstruction was the cross-plane Fuchs-Sondheimer model presented in Eq. (4). In **Figure S1*a*** it is easy to see that the different values of μ have an impact mainly in the accumulation function, being the case of the lower value of μ that affects the most both the accumulation function and the recovered LSSE, as shown in Figure S1***b***. The reconstructed function for the optimal value μ_{opt} = 0.8856 at T=250 K is shown in Fig. 10. The optimal value of the reconstruction parameter was calculated for all the temperatures measured, obtaining a different value for each of them (see **Figure S2**).

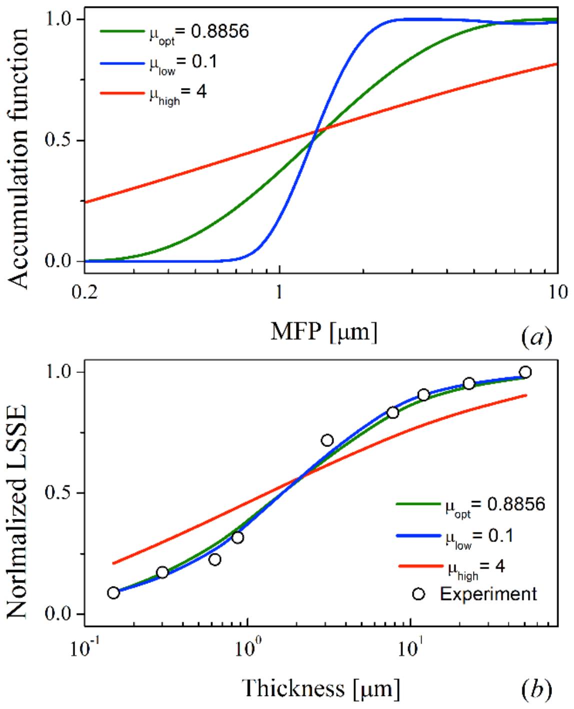

Figure S1 ***(a)*** *Magnon mean free path distribution of the accumulation function reconstructed for m_{opt} (green line) for spin Seebeck effect experiments. The blue and red lines are the result obtained using a low and high value of m, respectively. (b) Normalized Longitudinal Spin-Seebeck coefficient for different thickness of the YIG sample.*[1]

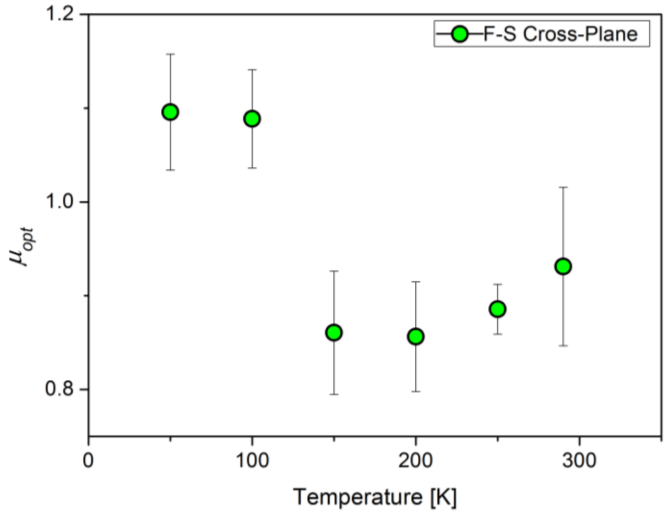

Figure S2 *Optimal values of μ for the Magnon-MFP reconstruction at different temperatures for LSSE experiments.*

Spin diffusion length in Pt films

This case is an example of the application of this technique to a completely different transport phenomena, namely, the spin diffusion length. The experiment consisted of measuring the thickness dependence of the spin-Hall torque coefficient, ξ, in platinum.[2]
In this case, the suppression function is derived from the drift-diffusion model [3], and given by [4]

$$S(\chi) = 1 - \frac{1}{\sinh(1/\chi)} \tag{S1}$$

This case differs from the previous phenomena, having a narrow span of the MFP distribution. For the optimal value of μ, the accumulation function's range goes from around 0.7 to 2 nm, and for μ = 0.1 it shrinks to be from 1 to 2 nm (see Figure S3). This indicates that the method is very sensitive even for very narrow MFP distributions, which allows it to be applied to a wide range of transport phenomena.

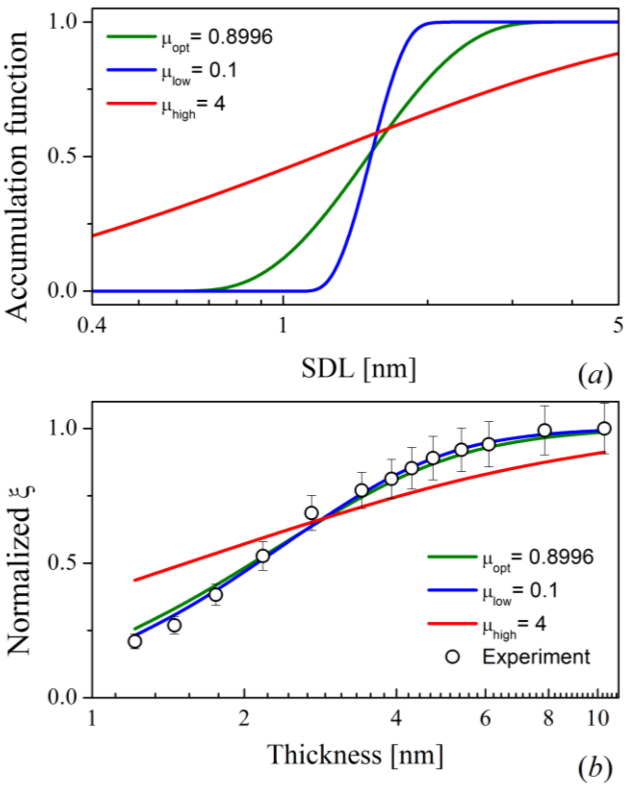

Figure S3 *Spin diffusion length distribution of the accumulation function reconstructed for spin-Hall torque experiments. The blue and red lines are the result obtained using a low and high value of μ, respectively. The green line represent the MFP distribution reconstructed using μ_{opt}. (b) Normalized Longitudinal spin-Hall torque coefficient for different thickness of the Pt filmvalues of μ for the Magnon-MFP reconstruction at different temperatures for LSSE experiments.*

References

[1] E.-J. Guo, J. Cramer, A. Kehlberger, C.A. Ferguson, D.A. MacLaren, G. Jakob et al., "*Influence of thickness and interface on the low-temperature enhancement of the spin Seebeck effect in YIG films*", Phys. Rev. X, vol. 6, no. 3, pp. 31012, 2016. DOI: 10.1103/PhysRevX.6.031012.

[2] M.-H. Nguyen, D.C. Ralph and R.A. Buhrman, "*Spin torque study of the spin Hall conductivity and spin diffusion length in platinum thin films with varying resistivity*", Phys. Rev. Lett., vol. 116, no. 12, pp. 126601, 2016. DOI: 10.1103/PhysRevLett.116.126601.

[3] P.M. Haney, H.-W. Lee, K.-J. Lee, A. Manchon and M.D. Stiles, "*Current induced torques and interfacial spin-orbit coupling: Semiclassical modeling*", Phys. Rev. B, vol. 87, no. 17, pp. 174411, 2013. DOI: 10.1103/PhysRevB.87.174411.

[4] A. Kehlberger, U. Ritzmann, D. Hinzke, E.-J. Guo, J. Cramer, G. Jakob et al., "*Length scale of the spin Seebeck effect*", Phys. Rev. Lett., vol. 115, no. 9, pp. 96602, 2015. DOI: 10.1103/PhysRevLett.115.096602.
